# Genome-wide identification, phylogenetic analysis, expression profiling, and protein–protein interaction properties of *TOPLESS* gene family members in tomato

**DOI:** 10.1093/jxb/ert440

**Published:** 2014-01-07

**Authors:** Yanwei Hao, Xinyu Wang, Xian Li, Carole Bassa, Isabelle Mila, Corinne Audran, Elie Maza, Zhengguo Li, Mondher Bouzayen, Benoit van der Rest, Mohamed Zouine

**Affiliations:** ^1^University of Toulouse, INPT, Laboratory of Genomics and Biotechnology of Fruit, Avenue de l’Agrobiopole BP 32607, Castanet-Tolosan F-31326, France; ^2^INRA, UMR990 Génomique et Biotechnologie des Fruits, Chemin de Borde Rouge, Castanet-Tolosan, F-31326, France; ^3^Laboratory of Fruit Quality Biology, The State Agriculture Ministry Laboratory of Horticultural Plant Growth, Development and Quality Improvement, Zhejiang University, Zijingang Campus, Hangzhou, PR China; ^4^Université de Toulouse, UPS, UMR 5546, Laboratoire de Recherche en Sciences Végétales, Castanet-Tolosan, France; ^5^CNRS, UMR 5546, 31326 Castanet-Tolosan, France; ^6^School of Life Sciences, Chongqing University, Chongqing 400044, China

**Keywords:** Aux/IAA, auxin signalling, co-repressor, multigenic family, protein–protein interactions, *Solanum lycopersicum*, tomato, TOPLESS.

## Abstract

Members of the *TOPLESS* gene family emerged recently as key players in gene repression in several mechanisms, especially in auxin perception. The *TOPLESS* genes constitute, in ‘higher-plant’ genomes, a small multigenic family comprising four to 11 members. In this study, this family was investigated in tomato, a model plant for Solanaceae species and fleshy fruits. Six open reading frames predicted to encode topless-like proteins (SlTPLs) containing the canonical domains (LisH, CTLH, and two WD40 repeats) were identified in the tomato genome. Nuclear localization was confirmed for all members of the SlTPL family with the exception SlTPL6, which localized at the cytoplasm and was excluded from the nucleus. *SlTPL* genes displayed distinctive expression patterns in different tomato organs, with *SlTPL1* showing the highest levels of transcript accumulation in all tissues tested except in ripening fruit where *SlTPL3* and *SlTPL4* were the most prominently expressed. To gain insight into the specificity of the different TOPLESS paralogues, a protein–protein interaction map between TOPLESS and auxin/indole-3-acetic acid (Aux/IAA) proteins was built using a yeast two-hybrid approach. The PPI map enabled the distinction of two patterns: TOPLESS isoforms interacting with the majority of Aux/IAA, and isoforms with limited capacity for interaction with these protein partners. Interestingly, evolutionary analyses of the *TOPLESS* gene family revealed that the highly expressed isoforms (*SlTPL1*, *SlTPL3*, and *SlTPL4*) corresponded to the three *TPL*-related genes undergoing the strongest purifying selection, while the selection was much weaker for *SlTPL6*, which was expressed at a low level and encoded a protein lacking the capacity to interact with Aux/IAAs.

## Introduction

It is now well accepted that transcriptional co-repressors play crucial roles in a broad range of plant developmental processes (Liu and Karmarkar, 2008; Krogan and Long, 2009). In land plants, the Groucho (Gro)/Tup1 family of co-repressors includes TOPLESS/TOPLESS-RELATED (TPL/TPR) and LEUNIG/LEUNIG HOMOLOG (LUG/LUH) (Conner and Liu, 2000; Kieffer *et al.*, 2006; Long *et al.*, 2006). TPL proteins have been shown to be involved in multiple signalling pathways in higher plants, including hormone-signalling pathways (auxin, jasmonic acid, abscisic acid, and ethylene), meristem maintenance, floral induction, biotic stress, and circadian oscillator mechanism (Liu and Karmarkar, 2008; Szemenyei *et al.*, 2008; Pauwels *et al.*, 2010; Zhu *et al.*, 2010, Causier *et al.*, 2012*a*, *b*; Wang *et al.*, 2013).

The first *TPL* gene was identified in *Arabidopsis* as responsible for the semi-dominant *tpl-1* embryo development mutation resulting in altered polarity, ranging from fused cotyledons to complete replacement of the shoot with a second root (Long *et al.*, 2002, 2006). Subsequently, the *TPL* family in *Arabidopsis thaliana* was found to comprise five members that seem to act redundantly (*TPL*, *TPR1*, *TRP2*, *TRP3*, and *TRP4*). Indeed, a quintuple loss of function, in which all five *TPL*/*TPR* genes were inactivated by mutation or RNA interference, is required to phenocopy the *tpl-1* phenotype (Long *et al.*, 2006).

It was established that, although TPL proteins are lacking a DNA-binding activity, they are incorporated into transcription complexes by interacting with transcription factors to repress gene expression in various processes. This inhibition of the expression of target genes is mediated by the recruitment of histone deacetylases into transcription complexes, and by changing the chromatin state from active to inactive (Long *et al.*, 2006; Liu and Karmarkar, 2008; Krogan and Long, 2009; Krogan *et al.*, 2012). Interaction between the TPL/TPR co-repressors and transcription factors depends on the Lissencephaly (LisH) and the C-terminal to LisH Homology (CTLH) domain of TPL (Szemenyei *et al.*, 2008; Gallavotti *et al.*, 2010), and on a small conserved protein motif found in transcription factors. This motif is known as the ethylene response factor-associated amphiphilic repression (EAR) domain (Ohta *et al.*, 2001), with the consensus sequence (L/F)DLN(L/F)xP (Ohta *et al.*, 2001; Hiratsu *et al.*, 2004). Recently, the *Arabidopsis* TPL/TPR interactome framework revealed that the TPL co-repressors are able to interact with various transcription factors harbouring different repression domains (Causier *et al.*, 2012*a*). Among these TPL interactants, the transcriptional repressors involved in auxin signalling [i.e. auxin/indole-3-acetic acid (Aux/IAA) and auxin response factor (ARF) families] have been well documented. In *Arabidopsis*, the discovery that TPL is recruited by Aux/IAA proteins to suppress the expression of auxin-responsive genes in the absence of auxin revealed a crucial role for TPL in mediating the inhibitory effect of Aux/IAA on ARF-regulated transcription (Szemenyei *et al.*, 2008). Large interactome studies in *Arabidopsis* identified 20 of the 29 *AtIAA* proteins as interacting partners of the TPL/TPRs (Arabidopsis Interactome Mapping Consortium, 2011; Causier *et al.*, 2012*a*). In addition, a large-scale analysis of the interaction between Aux/IAA and ARF in the *Arabidopsis* shoot apex revealed that the vast majority of the Aux/IAAs interacted with all the ARF activators and showed very limited interactions with ARF repressors (Vernoux *et al.*, 2011). However, a recent study showed that repressive ARF proteins, such as ARF2 and ARF9, can interact directly with TPL/TPR proteins, suggesting a mechanism for repression implicating TPL/TPR co-repressors in both forms of ARF-mediated repression (Causier *et al.*, 2012*a*).

The release in recent years of several plant genome sequences has offered the possibility to investigate a large set of multigenic families at the genome scale. In this context, the tomato genome is of special interest, as (1) tomato has emerged as a model plant, for fleshy fruit development, and (2) tomato is a reference species for the Solanaceae family and also for the taxum of Asterids, particularly as the majority of sequenced dicot genomes belongs to Rosids (Sato *et al.* 2012). It is noteworthy that the structure of several multigenic families involved in auxin perception and responses have been examined in tomato (Kumar *et al.*, 2011; Ren *et al.*, 2011; Wu *et al.*, 2011; Audran-Delalande *et al.*, 2012; Kumar *et al.*, 2012;Pattison & Catalá, 2012; Wu *et al.*, 2012*a*, *b*), thus shaping an exhaustive picture of auxin signalization complementary to the *Arabidopsis* model plant. However, compared with the plant model *Arabidopsis*, the *TPL* gene family has so far been poorly described.

To characterize fully the molecular biology and evolution of the tomato *TPL* family and to understand its possible functions, we identified and characterized six *SlTPL* genes. Our analyses focused on the identification, evolutionary relationships, and expression patterns of each member of the tomato *TPL* family. Moreover, we used yeast two-hybrid (Y2H) approaches to establish the framework of TPL/Aux/IAA protein–protein interactions (PPIs). These results will provide a framework for further studies to better understand the potential functions of TPL proteins in tomato plants, especially during the flower and fruit development.

## Materials and methods

### Isolation and cloning of *SlTPL* genes

The full-length coding sequences of six *SlTPLs* were amplified from mature green fruit cDNA. The primers used were as follows: TPL1_attb1: 5′-ATGTCATCTCTCAGTAGAG AGCTT-3′ and TPL1_attb2: 5′-TCATCTTGGTGCTTGATCGGAGC-3′; TPL2_attb1: 5′-ATGTCTTCCTTGAGTAGGGAACTG -3′ and TPL2_attb2: 5′-TCACCTTGAAGGTGTTTCTGATG-3′; TPL3_attb1: 5′-ATGTCTTCTCTTAGCAGAGAATTG-3′ and TPL3_attb2: 5′-TCATCTTTGAACTTGGTCAGCAG-3′; TPL4_attb1: 5′-ATGACTTCTTTAAGCAGAGAGCTG-3′ and TPL4_attb2: 5′-C TACCTTGATGCTTGATCAAGACC-3′; TPL5_attb1: 5′-ATG AGGCATTTTGATGAAATGGT-3′ and TPL5_attb2: 5′-CT ACCTTTGAGGTTGATCT GAAT-3′; and TPL6_attb1: 5′-ATGT CTCTTAGTAAGGACCTTAT-3′ and TPL6_attb2: 5′-CTATATTG GTTGCTCAT TGGTAA-3′.

After amplification, the *SlTPL* genes were cloned into the pDONOR207 vector using the Gateway method (Invitrogen) and were fully sequenced.

### Subcellular localization of SlTPL proteins

For localization of the SlTPL proteins, the *SlTPL* coding sequences were cloned using Gateway technology as a C-terminal fusion in frame with yellow fluorescent protein (YFP) into the pEarlyGate104 vector and expressed under the control of the 35S cauliflower mosaic virus promoter. The empty vector pEarleyGate104 was used as a control. Protoplasts were obtained from tobacco (*Nicotiana tabacum*) suspension-cultured BY-2-cells and transfected according to a method described previously (Leclercq *et al.*, 2005). YFP localization by confocal microscopy was performed as described previously (Audran-Delalande *et al.*, 2012).

### Expression analysis of *SlTPL* genes

Total RNA extraction, removal of DNA contamination, cDNA generation of eight tomato tissues (root, stem, leaves, bud, flower, mature green fruit, breaker fruit, and red fruit), and quantitative reverse transcription-PCR (qRT-PCR) were performed according to methods previously described (Audran-Delalande *et al.*, 2012; Pirrello *et al.*, 2006). The primer sequences were as follows: TPL1F: 5′-TGTTCGT TCTAGGAGACTAACCAG-3′ and 5′-TPL1R: AAGACAAACCTTCCCTTC CGA-3′; TPL2F: 5′-CC TGTAAATACGCCT CTTGCT-3′ and TPL2R: 5′-ACTGGTTGG AATGGACTGTG-3′; TPL3F: 5′-CACTTTCTGCTCCAATAA CCT-3′ and TPL3R: 5′-TCCA TCTGTCAACCCAACTG-3′; TPL4 F: 5′-CCTTCTAACC CAAGCTCCAG-3′ and TPL4R: 5′-AT AAACTCCGCCATCAGTA AGTC-3′; TPL5F: 5′-CGTCTATT GTAACCCATCCA CTC-3′ and TPL5R: 5′-AGAAGTTACACCAT GAGGACCC-3′; and TPL6F: 5′-ACTG GACTAGCATTCTCT AACAC-3′ and TPL6R: 5′-TTGAATT CCACA CCACTATCTG AG-3′. Actin was used as an internal reference. The relative fold differences (with *SlTPL6* as a reference gene) for each sample were calculated using the formula 2^–ΔΔ*C*t^. Three independent RNA isolations were used for cDNA synthesis and, each cDNA sample was subjected to real-time PCR analysis in triplicate.

### Bioinformatic analyses

*SlTPL* genes were searched using BLAST queries on the Genomic (Chromosome v.2.40) and transcript database (cDNA itag 2.4) available on the SGN website (http://solgenomics.net/tools/blast/index.pl). Exons and introns were deduced from the ITAG 2.3 annotation. For *SlTPL5* (Solyc07g008040), the ‘predicted annotation’ missing the N-terminal extremity was completed with an additional exon (from position 2754093 to 2754173 on SL2.40ch07 chromosome 7 annotation). Protein domains were first predicted on the prosite database protein (http://prosite.expasy.org/). Prediction of the WD40 segments was refined using the PF00400.27 Pfam Hidden Markov Model with an i-value threshold at 0.1. For i-values > 0.1, the prediction of WD40 position was deduced from the sequence alignment of the different *TPL* isoforms.

Nuclear localization signal (NLS) analysis prediction was performed with ‘cNLS Mapper’ (http://nls-mapper.iab.keio.ac.jp/cgi-bin/NLS_Mapper_form.cgi) (Kosugi *et al.*, 2009). NLS prediction scores >5.0 were considered positive.

### Evolutionary analyses

Phylogenetic analyses and distance matrices were built using the MEGA5 package (Tamura *et al.*, 2011). Full-length amino acid sequences were aligned using the ClustalW algorithm. For the overall phylogeny, an initial tree encompassing sequences from *Physcomitrella patens*, *Selaginella moellendorffii*, *Oryza sativa*, *Zea mays*, *Sorghum bicolor*, *Arabidopsis thaliana*, *Solanum lycopersicon*, *Nicotiana benthamiana*, *Populus trichocarpa*, *Glycine max* and *Mimulus guttatus* was performed using the neighbour-joining method. The percentage of replicate trees in which the associated taxa clustered together was calculated in the bootstrap test (500 replicates). The topology was further confirmed using the maximum-likelihood method. Ultimately, a simplified tree was performed by limiting the number of genomic sets as the topology remained unchanged. Trees were drawn to scale, with branch lengths in the same units as those of the evolutionary distances used to infer the phylogenetic tree.

The following genome annotations were used for phylogenetic analyses: *Physcomitrella patens* (Phypa1_1.FilteredModels; Rensing *et al.*, 2008); *Selaginella moellendorffii* (Lycophyte Selmo1_GeneModels_FilteredModels3; Banks *et al.*, 2011); *A. thaliana* (TAIR10; Swarbreck *et al.*, 2008); *Populus trichocarpa* (Eudicot Populus.trichocarpa.v2.0; Tuskan *et al.*, 2006); *V. vinifera* (12X March 2010 release, Glycine max Glyma1_pacId; Schmutz *et al.*, 2010); *O. sativa* [MSU Rice Genome Annotation (Osa1) Release 6.1; Ouyang *et al.*, 2007]; *Z. mays* (ZmB73_4a.53_working_translations; Schnable *et al.*, 2009); *Sorghum bicolor* (Sorbi1_GeneModels_Sbi1_4_aa; Paterson *et al.*, 2009); *Solanum lycopersicon* (ITAG2.3_release; Sato *et al.*, 2012); *Brassica rapa* (Chiifu-401–42; Wang *et al.*, 2011); *Eucalyptus grandis* (Egrandis_201; http://www.jgi.doe.gov/); *M. guttatus* (Mguttatus_140; http://www.jgi.doe.gov/); *N. benthamiana* (Niben.genome.v0.4.4; Pallas *et al.*, 2012); *Solanum tuberosum* (PGSC_DM_v3.4; Xu *et al.*, 2011).

### Protein–protein interaction (PPI) assay of SlTPLs and SlIAAs by Y2H assay

Tomato *TPL* genes were amplified and cloned into the pDBD (BD-TPLs) vector (Clontech). Similarly, *SlIAA* target genes [*IAA1* (JN379431), *IAA3* (JN379433), *IAA4* (JN379434), *IAA7* (JN379435), *IAA8* (JN379436), *IAA9* (JN379437), *IAA11* (JN379438), *IAA12* (JN379439), *IAA14* (JN379441), *IAA15* (JN379442), *IAA16* (JN379443), *IAA17* (JN379444), *IAA19* (JN379445), *IAA22* (JN379447), *IAA26* (JN379449), *IAA27* (JN379450) and *IAA29* (JN379451)] were inserted in pGAD (AD-IAAs) vectors (Clontech). Diploids were selected on medium lacking Trp and Leu, and interactions were validated by the use of *HIS3* and *ADE2* reporter genes on medium lacking Trp, Leu, His, and Ade. Manipulation and analysis of the Y2H assay followed the manufacturer’s instructions (Clontech Yeast Protocols Handbook), and all experiments were repeated three times independently. For *SlTPL1* genes lacking LisH, the coding sequence was truncated at nucleotide position +112.

## Results

### Identification and cloning of *TPL*-related genes in the tomato genome

An *in silico* search was performed on the tomato genome and transcript databases (http://www.solgenomics.net/) using Arabidopsis *TPL* and *TPR* sequences as queries for BLAST searches. While the initial screen identified nine ORFs predicted to encode putative TPL-like proteins (SlTPLs), only six corresponded to full-length proteins containing all canonical motifs that define the TPL proteins (Table 1). The full-length cDNA of the six *SlTPL*s was further confirmed by RT-PCR amplification, indicating that the corresponding coding sequences range from 3396 to 3669bp with deduced protein sizes ranging from 1131 to 1222 aa (Table 1).

**Table 1.** Main structural features of the tomato SlTPL family members

Nomenclature	Gene			Predicted protein		Domains		
SlTPLs	iTAG Gene ID	Exons	Introns	Length (aa)	MW (kDa)	LisH	CTLH	WD-40 repeats
SlTPL1	Solyc03g117360.2.1	25	24	1131	124.676	4–36	34–92	411–632/832/957
SlTPL2	Solyc08g076030.2.1	25	24	1136	124.60	4–36	34–92	341–668/834–959
SlTPL3	Solyc01g100050.2.1	25	24	1132	124.676	4–36	34–92	343–669/871–955
SlTPL4	Solyc03g116750.2.1	26	25	1133	124.318	4–36	34–92	413–634/839–964
SlTPL5	Solyc07g008040.2.1	24	23	1134	124.82	4–36	34–92	398–639/881/965
SlTPL6	Solyc08g029050.2.1	33	32	1222	134.181	3–35	33–91	531–664/934–1060

**Fig. 1.**
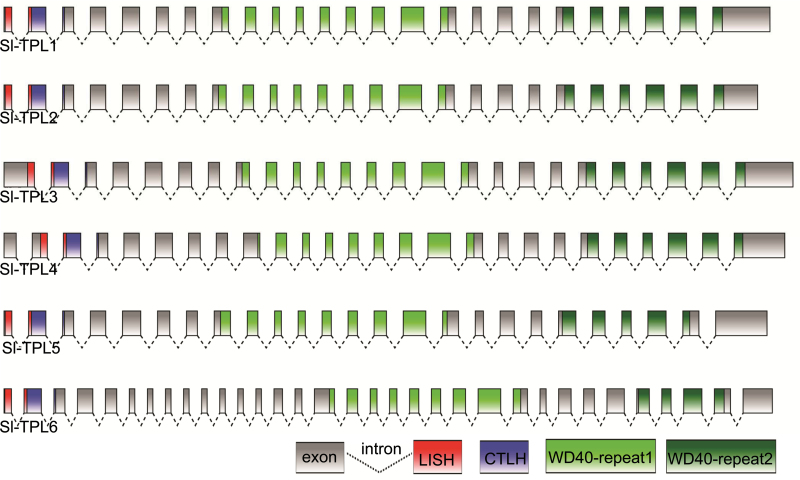
Gene structure of the six tomato *TPL* genes. Grey boxes represent exons, dotted lines represent introns, the red box is the LisH domain, the blue box is the CTLH domain, the light green boxes are the WD40-repeat 1 and the dark green boxes are the WD40-repeat 2. The figure was produced using FancyGene software (http://bio.ieo.eu/fancygene/). (This figure is available in colour at *JXB* online.)

Structural analysis of the six *SlTPL* genes showed that they displayed similar numbers of introns (23–25) and exons (24–26), except for *SlTPL6*, which was longer than the other *TPL* members (Table 1). Pairwise comparison of the six SlTPL protein sequences showed that the percentage identity among family members ranged from 44 to 75%. Protein domain searches in the Pfam database (http://pfam.sanger.ac.uk/) indicated that all SlTPLs displayed the conserved LisH and CTLH domains and had two domains containing several WD40 repeats: WD40-repeat-1 and WD40-repeat-2 with seven and five WD40 segments, respectively (Fig. 1 and Supplementary Fig. S1 available at *JXB* online). The CTLH domain and the WD40-repeat-1 were separated by a proline-rich region.

The tomato *TPLs* were distributed on four chromosomes: two *SlTPL*s (Solyc03g116750 and Solyc3g117360) on chromosome 3, two (Solyc08g076030.2.1 and Solyc08g029050.2.1) on chromosome 8, one (Solyc01g100050.2.1) on chromosome 1 and one (Solyc07g008040.2.1) on chromosome 7. There were three additional truncated TPL sequences lacking the LisH and CTLH domains, with two located on chromosome 3 (Solyc03g117370 and Solyc03g117410) and one on chromosome 1 (Solyc05g016070).

**Fig. 2.**
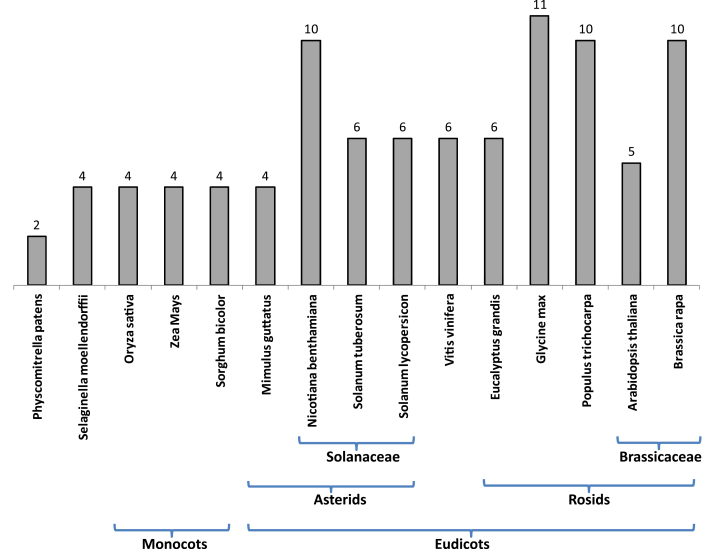
Inventory of *TPL* genes in different plant genomes. Only *TPL* genes containing the four canonical domains (LisH, CTLH and two WD40 repreats) were considered. The major taxons are shown below.

The number of ‘full-length’ *TPL* genes in tomato fell in the range found in other plant genomes, which varies in angiosperms from four members in monocots to 11 members in soybean (Fig. 2). It is noteworthy that a high number of isoforms is often observed in organisms having undergone recent whole-genome duplication or polyploidization events (e.g. *G. max*, *N. benthamiana* and *B. rapa*).

### SlTPL nomenclature and phylogenetic analyses

**Fig. 3.**
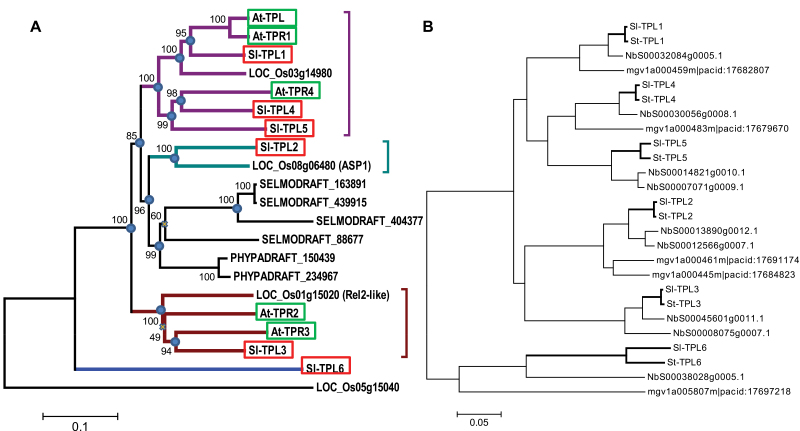
Phylogenetic trees of some plant and tomato TPL proteins. (A) Representative phylogenetic tree of TPL proteins from land plants: moss (*P. patens*, PHYPADRAFT_xxx), lycophyte (*Selaginella moellendorffii*, SELMODRAFT_xxx), rice (LOC_Os-xxx), tomato (red boxes) and *Arabidopsis* (green boxes). The coloured brackets emphasize the main branches conserved among angiosperms. The present tree was obtained after alignment of full-length TPL sequences using ClustalW and clustering with the neighbour-joining method. The percentages of replicate trees in which the associated taxa clustered together in the bootstrap test (500 replicates) are shown next to the branches. Phylogenetic analyses including additional genome sets (*Z. mays*, *Sorghum bicolor*, *Populus trichocarpa*, *G. max*, *V. vinifera* and *M. guttatus*) or using the maximum-likelihood clustering method displayed similar topologies, the majority of the nodes being conserved (blue circles) while only few nodes (yellow crosses) were unstable. (B) Phylogenetic tree of TPL proteins among Asterid and Solanaceaous species. The tree was built using sequences from four genomes: *Solanum lycopersicon*, *Solanum tuberosum*, *N. benthamiana* and *M. guttatus*. (This figure is available in colour at *JXB* online.)

To adopt a nomenclature consensual with that of *Arabidopsis* TPL and TPR proteins, we carried out phylogenetic analyses on different TPL-like proteins or cDNAs from different plant sequenced genomes comprising moss, fern, and various angiosperm sequences (see Materials and methods). The phylogenetic trees (Fig. 3A) allowed the individualization of four branches. Three branches looked well defined in all dicot plants: the first branch contained AtTPL, AtTPL1, AtTPR4, Solyc3g117360.2.1 (named SlTPL1), Solyc03g117360.2.1 (named SlTPL4), and Solyc07g008040.2.1 (named SlTPL5); the second branch, absent in *Arabidopsis* yet present in *Eucalyptus* (Eucgr.K00093.1|PACid:23601479) and grapes (GSVIVT01024440001), contained Solyc08g076030 (named SlTPL2), rice ASP1 protein, and moss or lycophyte TPL-like proteins; and the third branch contained AtTPR2, AtTPR3, and Solyc01g100050.2.1 (named SlTPL3). Lastly, Solyc08g029050.2.1 (named SlTPL6) appeared as an outgroup branch in the phylogenetic tree (Fig. 3A). The robustness of the tree topology was assessed either with a bootstrap test (Fig. 3A) or by changing the number of genomes used in the phylogeny and the portion of the aligned sequence (N-terminal, C-terminal, or conserved domains) or the clustering method (neighbour-joining or maximum-likelihood method). The vast majority of the nodes presented in Fig. 3A remained unchanged.

To understand further the TPL phylogeny, and notably to characterize the SlTPL6 outgroup, the presence of TPL ‘orthologues’ was investigated in Asterid genomes belonging either to the Solanaceae family (*Solanum tuberosum* and *N. benthamiana*) or to the Lamiales order (*M. guttatus*). An SlTPL6 homologue was found in all Asterids, supporting the view that SlTPL6 homologues form a distinct clade (Fig. 3B). Within this SlTPL6 clade, the length of the branches suggested that these isoforms had evolved faster than other TPLs. This observation was supported by sequence divergences: the amino acid substitution rates calculated within the Solanaceae orthology groups varied from 2.6 to 6.3% for SlTPL1–5 and reaching 22.7% for the SlTPL6 (Table 2). Moreover, a neutrality test (*dS*/*dN* values) calculated on Solanaceae orthologues suggested that the purifying selection exerted by evolution on the *SlTPL6* family is much weaker than the selection pressure exerted on other *TPL* genes.

**Table 2.** Evolutionary features of TOPLESS-related genes in Solanaceous speciesMean distance was expressed as the proportion of amino acid or nucleic acids positions different after pairwise alignment. *dS*/*dN* values were calculated using the codon-based test of purifying selection performed on each pair of orthologous sequences from *Solanum lycopersicon* and *Solanum tuberosum.* The variance of the difference was computed using the bootstrap method (500 replicates). Analyses were conducted using the Nei and Gojobori (1986) method.

		SlTPL1	SlTPL2	SlTPL3	SlTPL4	SlTPL5	SlTPL6
Mean distance (*Solanum*/*Nicotiana*)	Amino acids	0.026	0.041	0.032	0.029	0.063	0.227
	Nucleic acids	0.055	0.050	0.054	0.054	0.067	0.154
Neutrality test (*Solanum*)	*dS*/*dN*	7.08	6.66	6.98	7.62	6.19	3.645

### Subcellular localization of SlTPLs

**Fig. 4.**
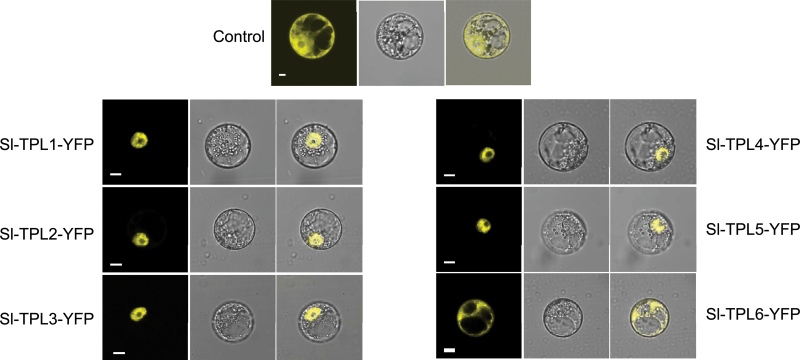
Subcellular localization of tomato TPL proteins. SlTPL–YFP fusion proteins were transiently expressed in BY-2 tobacco protoplasts and subcellular localization was analysed by confocal laser-scanning microscopy. The merged pictures of the yellow fluorescence channel (left panels) and the corresponding bright field (middle panels) are shown (right panels). The empty vector pEarleyGate104 was used as a control. Bar, 10 μm. (This figure is available in colour at *JXB* online.)

The subcellular localization of the SlTPL proteins was assessed by a transient expression assay in tobacco protoplasts using a translational fusion between each of the SlTPL proteins and YFP. Microscopy analysis showed that SlTPL1–5–YFP fusion proteins localized exclusively to the nucleus (Fig. 4) whereas SlTPL6 was localized at the cytoplasm and excluded from the nucleus. This result is in agreement with the *in silico* prediction of a conserved NLS for the five nuclear SlTPL1–5 proteins, while SlTPL6 NLS scores were below the 5.0 threshold value (Supplementary Table S1 available at *JXB* online). Altogether, the nuclear localization of the majority of SlTPLs was consistent with their putative role in transcriptional regulation activity.

### Expression analyses

**Fig. 5.**
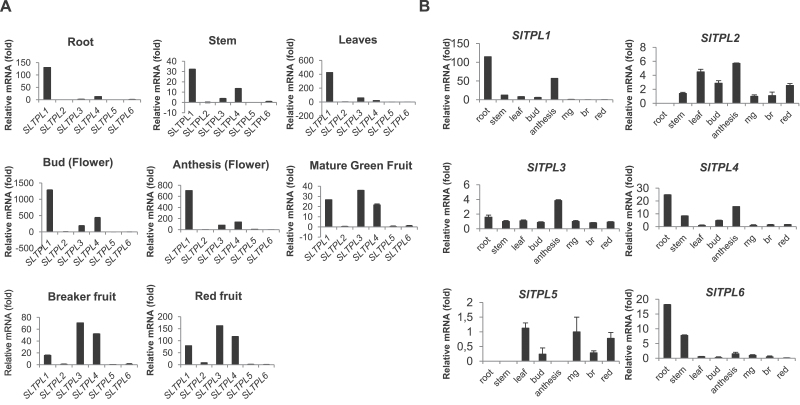
Real-time PCR expression profiles of six tomato *TPL* genes. (A) Expression patterns of *SlTPL* genes in various tomato tissues. Relative mRNA levels of each *SlTPL* gene in different tissues were normalized against actin. The results were expressed using *SlTPL6* as a reference (relative mRNA level 1). Values represent the best experiment among three independent biological repetitions. Bars indicate the standard deviation of three experimental repetitions. (B) Expression patterns in different tomato tissues of each *SlTPL* gene. The relative mRNA level of each *SlTPL* gene was normalized against actin. mg, Mature green fruit; br, breaker fruit; red, red fruit. The results were expressed using the mature green fruit as a reference (relative mRNA level 1). Values represent the best experiment among three independent biological repetitions. Bars indicate the standard deviation of three experimental repetitions.

In order to study the spatio-temporal expression pattern of the six *SlTPL* genes, qRT–PCR was performed on eight different plant tissues and organs. Three *SlTPL* members (*SlTPL1*, *SlTPL3*, and *SlTPL4*) displayed significantly higher levels of expression than the three remaining paralogues. *SlTPL1* and *SlTPL4* were found to be highly expressed in flowers and vegetative tissues (roots, stems, and leaves) and in developing flowers (buds and during anthesis) but with reduced expression in ripening fruit, while *SlTPL3* expression remained constant and high during fruit ripening (Fig. 5). This preferential expression of *SlTPL1*, *SlTPL3*, and *SlTPL4* is coherent with their estimated expression in two public databases (RNAseq database: http://ted.bti.cornell.edu and ESTs database: http://solgenomics.net/). Although less expressed, *SlTPL2* was found preferentially in leaves and developing flowers; the levels of *SlTPL5* transcripts were low in all tissues; *SlTPL6* expression was restricted to roots and stems (Fig. 5b).

### Examination of PPIs in the framework of auxin mediation

**Fig. 6.**
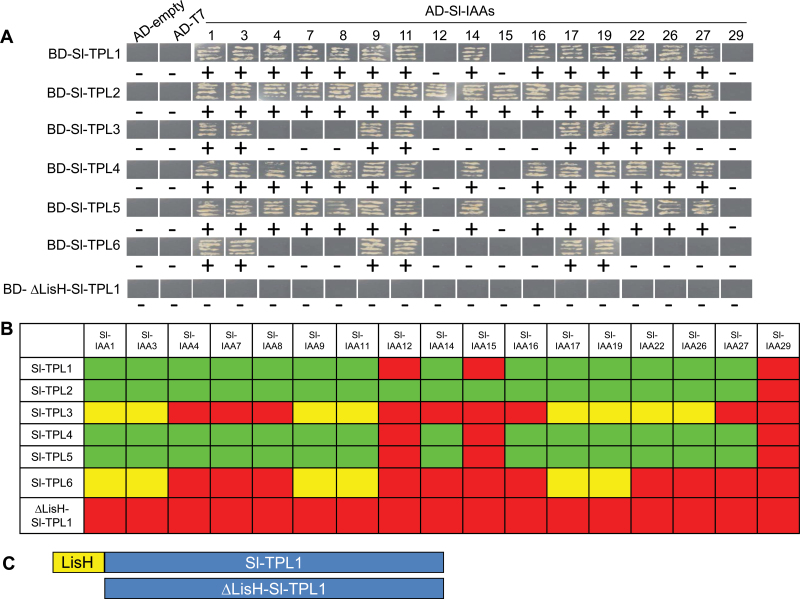
PPI maps between SlTPLs and SlIAAs established by a Y2H screen. (A) Yeast growth of co-transformed BD–TPLs and AD–IAAs. The yeast clones grown on selected medium lacking Trp, Leu, His, and Ade (TLHA) were scratched again on a TLHA plate. After 3–4 d, the growth of the yeast strains confirmed a positive interaction, as shown. AD–empty vector and AD–T7 vector were used as negative controls. (B) Schematic representation of the interaction map between SlTPLs and SlIAAs. Green indicates that the yeast grew quickly, less than 4 or 5 d after co-transformation, indicating a strong interaction between the SlTPL and SlIAA partners. Yellow indicates that the yeast grew slowly 7–8 d after co-transformation, indicating a weak interaction between the tested SlTPL and SlIAA. Red indicates that there was no interaction detected between the tested SlTPLs and SlIAAs. (C) Truncated form of SlTPL1 protein lacking the N-terminal LisH domain N-terminal used as a negative control. (This figure is available in colour at *JXB* online.)

The differential expression of *SlTPL* genes evokes the critical question of functional redundancy within the TPL family. In a recent paper, Causier *et al.* (2012*a*) compared the PPI patterns of different *Arabidopsis* TPL proteins using a high-throughput Y2H screen both on a whole-plant and on a transcription factor library. In the present work, we focused on the interactions with the Aux/IAA family by performing an exhaustive targeted analysis of Aux/IAA–TPL interactions. The six SlTPL proteins were fused to a binding domain (BD) and used as bait in a Y2H test with 17 different SlIAA proteins fused to an activating domain (AD). After monitoring the yeast growth on two auxotroph selective media, two patterns of TPL could clearly be defined (Fig. 6A, B): SlTPL1, SlTPL2, SlTPL4, and SlTPL5 interacted with the majority of SlIAAs and grew in all the selective media, and SlTPL3 and SlTPL6 exhibited only limited growth when co-expressed with Aux/IAA–AD fusion proteins. Contrary to other SlIAAs, SlIAA29 failed to show interaction with any of the SlTPLs. With the exception of SlIAA12 and SlIAA15, the Aux/IAAs did not harbour any obvious specificity towards the ‘TPL’ clade (SlTPL1, SlTPL4, and SlTPL5), sharing high similarity with AtTPL. In addition, SlTPL2, which belongs to a distinct clade of SlTPLs (1, 4, and 5), also exhibited a broad capacity to interact with the majority of SlIAAs. As a control, we performed a Y2H test with truncated SlTPL1 or SlTPL5 (ΔLisH-TPL) (Fig. 6C) lacking the LisH domain shown previously to be essential for TPL–WUS or TPL–Aux/IAA interactions (Kieffer *et al.*, 2006; Szemenyei *et al.*, 2008). Contrary to all SlTPLs BD fusions assayed, a complete lack of growth was observed when co-expressing BD–ΔLisH-TPL proteins with BD–Aux/IAAs (Fig. 6B).

## Discussion

The present study addressed the structural, evolutionary, and functional features of the tomato *TPL* family. TPL proteins have been primarily defined as a major component of the auxin transduction and response pathway, but the present data sustain the hypothesis of a functional diversification of these regulatory proteins. While mainly focusing on the *TPL* family in tomato, a plant model for Solanaceae and fleshy fruit research, the data also addressed the comparative features of this gene family within Plant kingdom at the evolutionary level, shedding new light on their functional diversification.

The structure of the *SlTPL* family is representative of that found in angiosperms where these proteins belong to a small multigenic family comprising five to 11 members. In the tomato, six full-length *SlTPL* genes were identified, as well as additional three pseudogenes with incomplete coding sequences. Among the six *SlTPL* genes, five were highly conserved (*SlTPL1–5*), while the last gene (*SlTPL6*) was more distant. With the exception of poplar genomes and genomes having undergone recent polyploidization (i.e. soybean, *B. rapa*, and *N. benthamiana*), the number of *TPL* isoforms ranges from four to six members, suggesting that the number of genes remains stable in this family and that usually, after a whole-genome duplication event, duplicated copies of *TPL* genes are not retained. The phylogenetic analysis of *TPL* genes enabled the distinction of three major clades gathering homologues in the majority of angiosperm genomes. The last clade, containing the distant *SlTPL6*, displays only clear homologues in closely related taxa (Asterids). Interestingly, highly diverging sequences of TPL-related proteins have also been found in other genomes such as the *AtTPR-like* gene (At2g25420; Causier *et al.*, 2012b) and in poplar, but no clear relationship could be established with *SlTPL6*. Contrary to angiosperm TPL proteins, TPL from *Physcomitrella patens* and *Selaginella moellendorffii* clustered in a same branch, indicating the existence of ancestral divergences occurring before angiosperm radiation.

The functionality of *SlTPL* genes was addressed through three approaches: expression analysis, subcellular localization, and establishment of an interaction map between SlTPL and SlIAA proteins. The expression patterns of different *SlTPLs* revealed the tissue specificity of various isoforms and suggested a functional specialization of *SlTPL* isoforms. For example, *SlTPL1* is highly expressed in vegetative organs (stems, roots) and flowers, while the expression of *SlTPL3* and *SlTPL4* prevails in fruit. Moreover, the overall intensity of gene expression evaluated by qPCR demonstrated a distinction between a group of three isoforms (*SlTPL1*, *SlTPL3*, and *SlTPL4*) that are highly expressed, *SlTPL2*, which is moderately expressed in the leaves and flowers, and third group made of two isoforms (*SlTPL5* and *SlTPL6*) that displayed very low levels of expression. In agreement with our data, the prevalence of *SlTPL1*, *SlTPL3*, and *SlTPL4* transcripts was also observed in expressed sequence tag (EST) and RNAseq expression databases (http://ted.bti.cornell.edu), whereas the expression of *SlTPL6* was again found to be very low (no EST and few RNAseq reads). Interestingly, the overall expression level negatively correlated with the amino acid substitution rate. Indeed, after defining orthology groups among *Solanaceous TPLs*, we found that the highly expressed isoforms (*SlTPL1*, *SlTPL3*, and *SlTPL4)* showed the highest amino acid sequence conservation (<3.2% difference within *Solanaceous* sequences), while sequences were less conserved within the SlTPL6 orthology group (22.7% difference within *Solanaceous* sequences). The moderately expressed *SlTPL2* and *SlTPL5* displayed intermediate substitution rates (4 and 6% differences, respectively). This correlation was also supported by a neutrality test (*dS*/*dN* values) performed between potato and tomato pairs of othologues. The high substitution rate within the *SlTPL6* orthology group was interpreted as an indication that the *SlTPL6* subfamily undergoes a reduced purifying selection. By contrast, broadly expressed *SlTPL* isoforms are under a stronger purifying selection. Such a correlation between gene expression level and amino acid substitution rate has already been observed in genome-wide comparisons of expression patterns and protein evolution in *Arabidopsis*-related plants and in the Poaceae family (Wright *et al.*, 2004; Slotte *et al.*, 2011; Davidson *et al.*, 2012). Indeed, this correlation is consistent with *A. thaliana* expression data (AtGenExpress), *At-TPL* being expressed more than other *AtTPR*s and *AtTPL* orthologues remaining highly conserved either in *Arabidopsis lyrata* or in *B. rapa*.

The subcellular localization established a second discrimination criterion among SlTPLs. YFP fusion proteins of SlTPL1–5 isoforms all migrated exclusively to the nucleus, as observed with other TPL proteins from *Arabidopsis* (Long *et al.*, 2006), maize (Gallavotti *et al.*, 2010), and rice (Yoshida *et al.*, 2012). By contrast, the SlTPL6–YFP fusion protein displayed a divergent subcellular targeting, this isoform being targeted to the cytosol. This divergent localization is in line with the lower scores calculated by the NLS prediction tool for SlTPL6. This observation, in addition to the low expression level and the high substitution rate, supports the view of either a partial loss of functionality or divergent functionality regarding SlTPL6.

The first established function of TPL proteins is related to their role in auxin signalling via interaction with Aux/IAA partners (Szemenyei *et al.*, 2008). To check whether this role is conserved among all SlTPLs isoforms and gain insight on either functional redundancy or potential functional diversification among family members in tomato, a comprehensive PPI study was carried out between all SlTPLs and SlIAA members using a Y2H screen. This targeted interactome study revealed two distinct patterns of interaction for tomato TPLs: four isoforms (SlTPL1, SlTPL2, SlTPL4, and SlTPL5) displayed a broad capacity for interaction with the majority of SlIAAs, and the remaining two isoforms (SlTPL3 and SlTPL6) showing a more restricted interaction capacity. It is noteworthy that a large number of SlIAAs showed positive interaction with SlTPLs, consistent with the outcome of Y2H screens performed in *Arabidopsis* where 20 out of the 29 AtAux/IAAs were able to interact with AtTPLs (Szemenyei *et al.*, 2008; Arabidopsis Interactome Mapping Consortium, 2011; Causier *et al.*, 2012*a*). Interestingly, neither SlIAA29 nor its *Arabidopsis* homologue At IAA29 (AT4G32280.1) interacted with TPL proteins, although SlIAA29 exhibits a repressor activity (Audran-Delalande *et al.*, 2012). On the other hand, the limited interaction capacity displayed by SlTPL6 adds another distinctive feature to this isoform, which has already diverged from other family members by its low expression level, high amino acid substitution rate and different subcellular localization. Altogether, the cumulative distinctive features support the idea that SlTPL6 has partially lost its ancestral function and may have gained new functionality.

In previous Y2H screens performed in *Arabidopsis* by Causier *et al.* (2012*a*), AtTPR3 and AtTPR2, closely related to SlTPL3, both displayed the capacity to interact with various Aux/IAA proteins. However, a closer look at the interaction map published by Causier *et al.* (2012*a*) could also suggest differences in specificity between AtTPL and AtTPR2 or AtTPR3, with the two latter notably interacting with partners displaying partial repression domains. Such hypothesis opens the possibility that At-TPR2, At-TPR3 and the closely related SlTPL3 display a specialization alternative to auxin signalling. The development of quantitative PPI methods such as Förster resonance energy transfer or surface plasmon resonance may provide deeper insight on discriminating interaction features among various TPL isoforms.

Functional redundancy among *Arabidopsis* TPL family members is supported by the absence of obvious phenotypes in single loss-of-function mutants of *AtTPL/TPR* genes and by the requirement for downregulation of all five *AtTPL-TPRs* in order to phenocopy the dominant mutation *tpl-1* (Long *et al.*, 2006). However, this assumption is in contrast to the situation prevailing in rice and maize, where genetic evidence seems to support a more specialized functionality for *TPL* genes. Thus, in rice (Yoshida *et al.*, 2012), a single recessive mutation in *Asp1*, a *TPL-like* gene close to *SlTPL2*, exhibited several pleiotropic phenotypes, such as altered phyllotaxy and spikelet morphology. While these phenotypes suggest a close association of *Asp1* with auxin action, they clearly reveal that the specialization of TPL-related proteins in some organisms can differ from that in *Arabidopsis*. Further evidence sustaining a diversified function for TPL proteins is provided by maize *rel2* mutants affected in a *TPL-like* gene closely related to *SlTPL3* and *AtTPR3* (Gallavotti *et al.*, 2010). A better clarification of the putative specialized functionality among tomato TPLs might be addressed by a reverse genetics approach. Simultaneous downregulation of *SlTPL1* and *SlTPL4* would uncover the importance of the TPL family in vegetative development and auxin action. Likewise, specific downregulation of *SlTPL3* would be of particular interest to unravel the role of TPL co-repressors in flower and fruit biology.

Altogether, these data shed new light on structural, evolutionary, and some functional features of the tomato *TPL* gene family that suggest functional diversification of these regulatory proteins. Of particular interest, the setup of a comprehensive TPL–Aux/IAA interaction map and the differential subcellular targeting of some SlTPLs proteins would provide important clues towards designing appropriate strategies for the elucidation of both redundant and specific roles of *TPL* genes.

## Supplementary data

Supplementary data are available at *JXB* online.

Supplementary Fig. S1. Multiple sequence alignment of full-length SlTPL proteins.

Supplementary Table S1. NLS prediction scores computed with cNLS Mapper (Kosugi *et al.*, 2009).

Supplementary Data

## Abbreviations:

AD: activating domain
ARF: auxin response factor
Aux/IAA: auxin/indole-3-acetic acid
BD: binding domain
EST: expressed sequence tag
NLS: nuclear localization signal
PPI: protein-protein interaction
qRT-PCR: quantitative reverse transcription-PCR
Y2H: yeast two-hybrid
YFP: yellow fluorescent protein.
