# Supplementary material for: Genome-wide identification, phylogenetic analysis, expression profiling, and protein–protein interaction properties of TOPLESS gene family members in tomato
Source: J Exp Bot. 2014 Jan 7;65(4):1013–23. doi: 10.1093/jxb/ert440 (PMC3935560; doi:10.1093/jxb/ert440)
Supplement: Supplementary Data [file supp_65_4_1013__index.html]

Genome-wide identification, phylogenetic analysis, expression profiling, and protein–protein interaction properties of TOPLESS gene family members in tomato — Genome-wide identification, phylogenetic analysis, expression profiling, and protein–protein interaction properties of TOPLESS gene family members in tomato — Supplementary Data 

# Genome-wide identification, phylogenetic analysis, expression profiling, and protein–protein interaction properties of *TOPLESS* gene family members in tomato

## Supplementary Data

Data files

**Files in this Data Supplement:**

- Supplementary Data - Supplementary Data
