# Supplementary material for: Genome-wide identification, phylogenetic analysis, expression profiling, and protein–protein interaction properties of TOPLESS gene family members in tomato
Source: J Exp Bot. 2014 Jan 7;65(4):1013–23. doi: 10.1093/jxb/ert440 (PMC3935560; doi:10.1093/jxb/ert440)
Supplement: Supplementary Data [file supp_ert440_jexbot110148_file001.pdf]

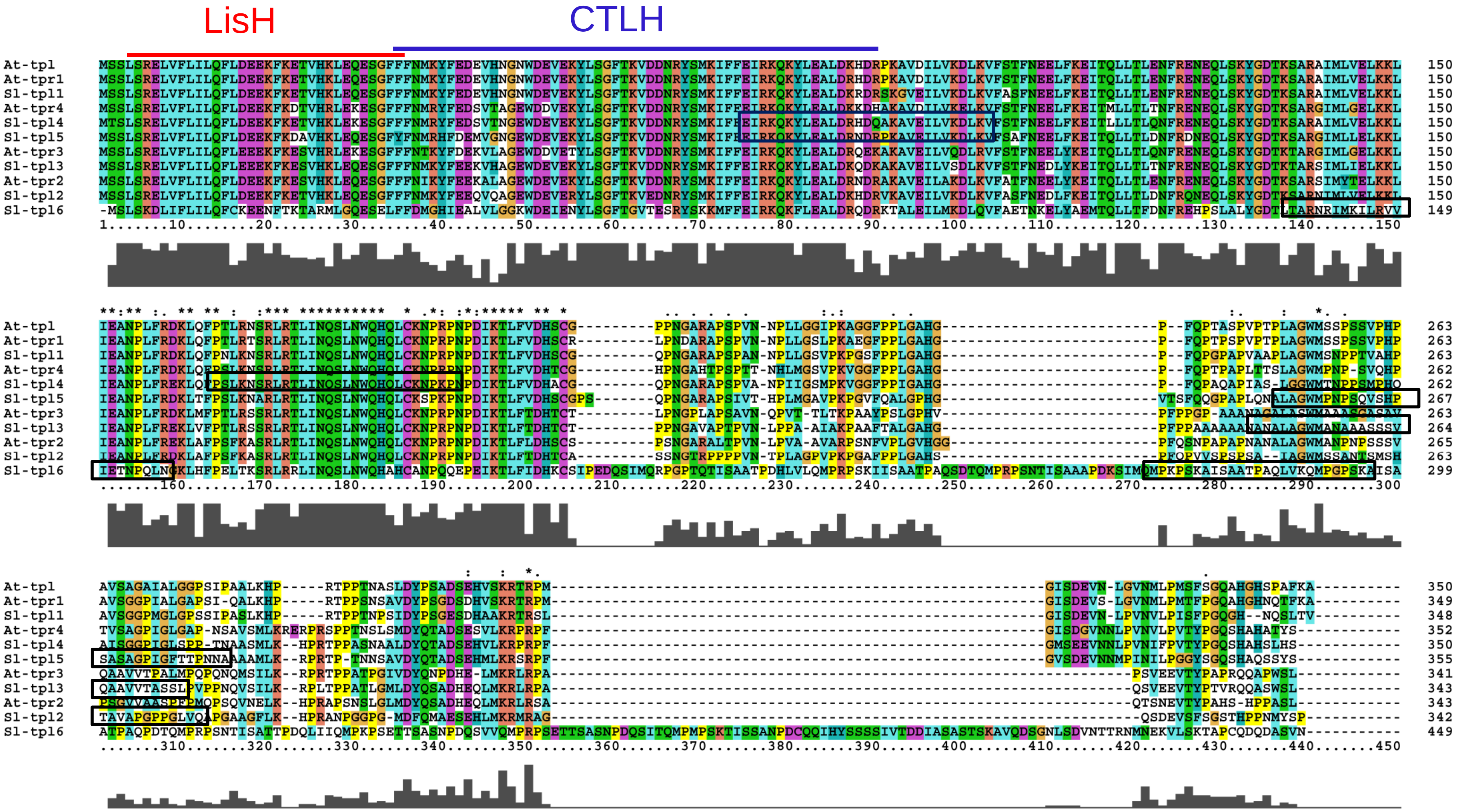

Supplementary Figure S1. Multiple sequence alignment of full-length Sl-TPL proteins. Conserved domains are underlined. The red line represents LisH domain. The blue line represents CTLH domain. The light green line represents the WD40-repeat 1 domain, the dark green line represents WD40-repeat 2 domain.

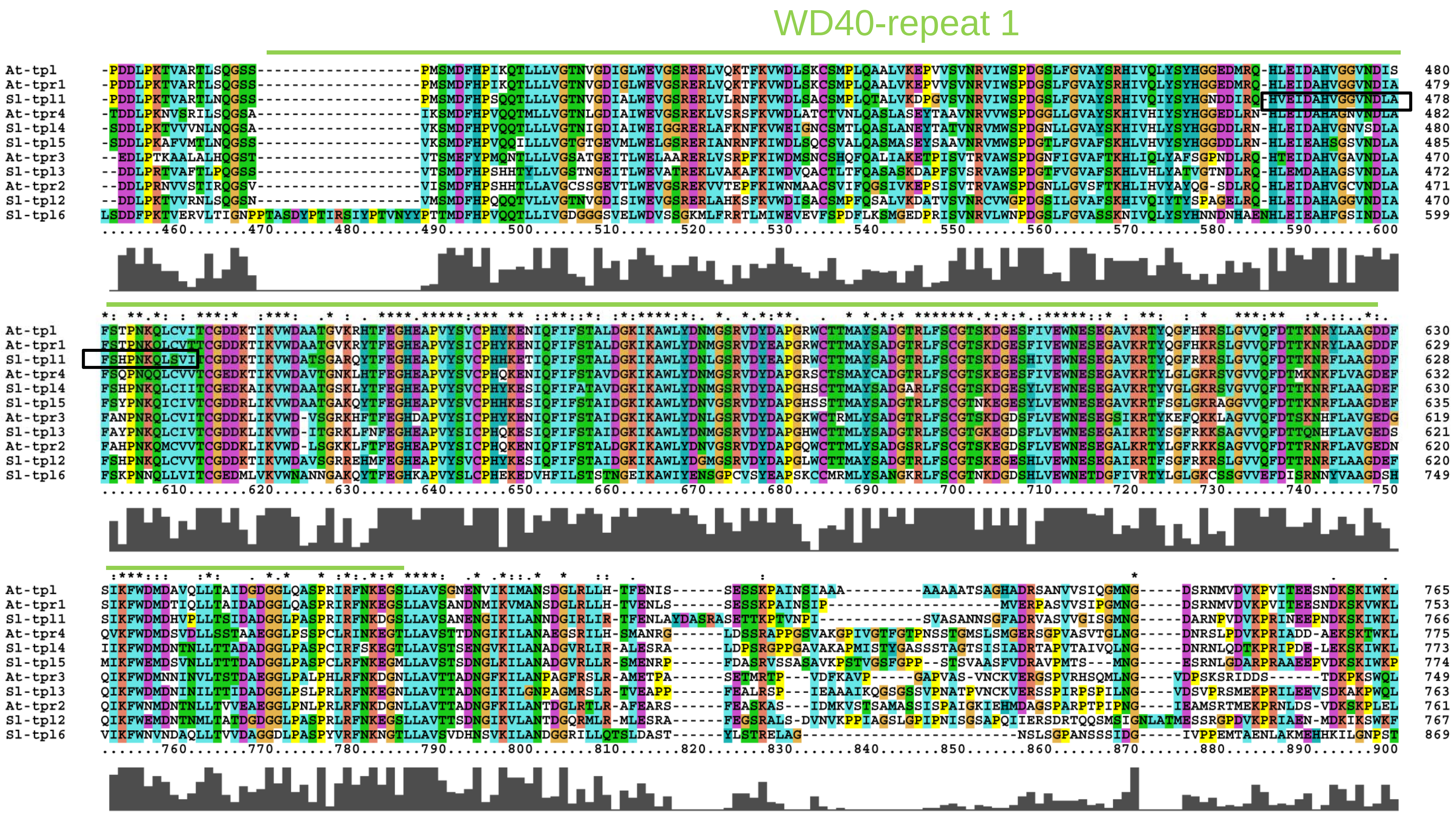
WD40-repeat 1
At-tpl
At-tpr1
Sl-tpl1
At-tpr4
Sl-tpl4
Sl-tpl5
At-tpr3
Sl-tpl3
At-tpr2
Sl-tpl2
Sl-tpl6

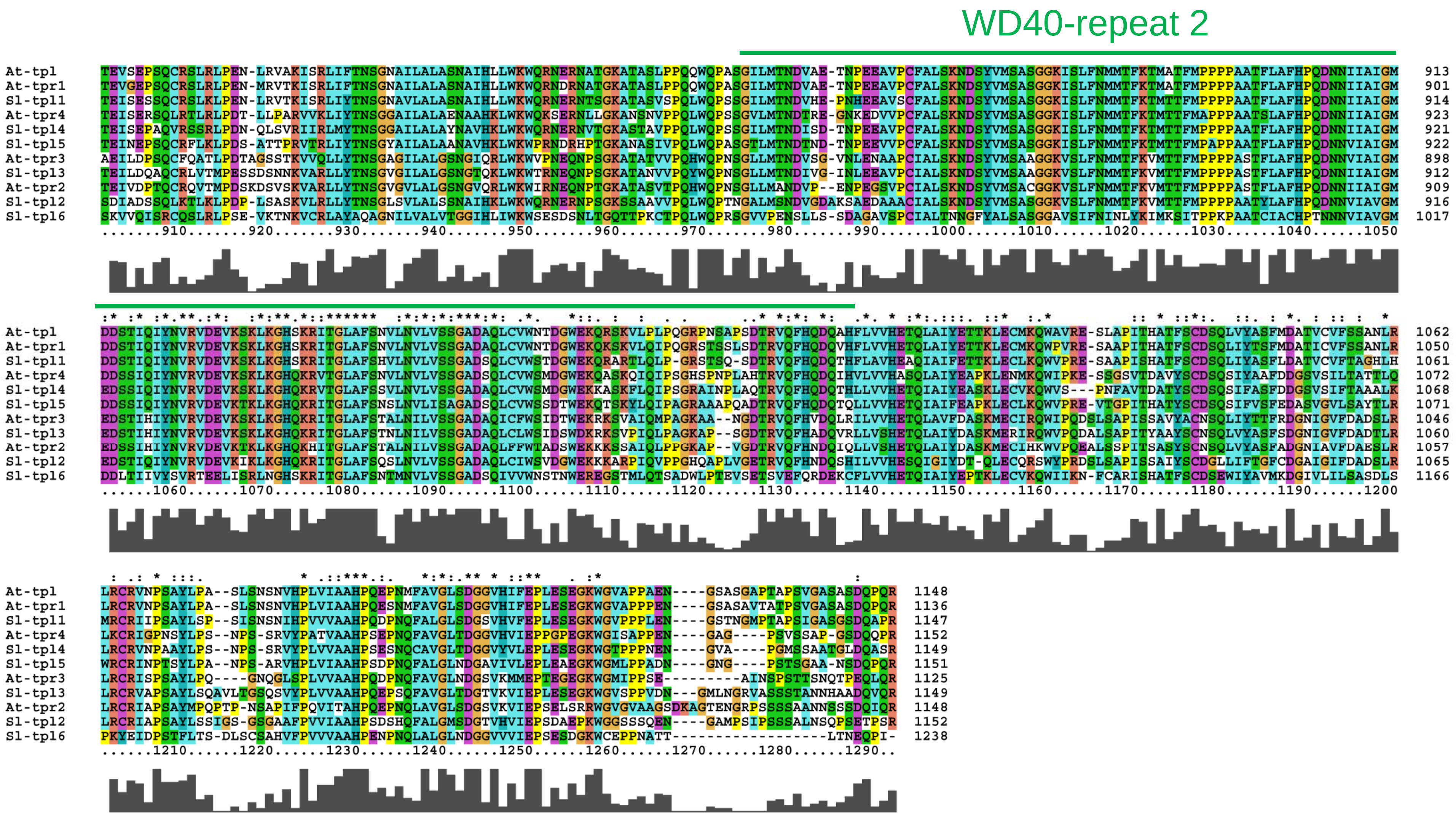
WD40-repeat 2
At-tpl
At-tpr1
Sl-tpl1
At-tpr4
Sl-tpl4
Sl-tpl5
At-tpr3
Sl-tpl3
At-tpr2
Sl-tpl2
Sl-tpl6

Supplementary Table 1 : NLS prediction scores computed with cNLS Mapper (Kosugi et al, 2009)

Sl-TPL	Score	Position	NLS sequence	Structure
**Sl-TPL1**	6	588	RTYQGFRKRSLGVVQFDTTKNRFL	Bipartite
**Sl-TPL2**	5	304	HLMKRMRAG	Monopartite
**Sl-TPL3**	5.8	283	ILKRPLTPPATLGMLDYQSADHEQLMKRL	Bipartite
**Sl-TPL4**	5.3/ 5	74	FEIRKQKYLEALDRHDQAKAVEILVKDLKV	Bipartite
		163	FPSLKNSRLRTLINQSLNWQHQLCKNPKP	
**Sl-TPL5**	5.2/5.7	-37	FEIRKQKYLEALDRNDRPKAVEILVKDLKV	Bipartite
		-249	MLKRPRTPTNNSAVDYQTADSEHMLKRSRP	
**Sl-TPL6**	4.1/ 4.2	-139	RNRIMKILRVVIETNPQLNGKLHFPELTKSRL	Bipartite
		-271	MPKPSKAISAATPAQLVKQMPGPSKAISA	
